# The improved antitumor efficacy of continuous intratumoral chemotherapy with cisplatin-loaded implants for the treatment of sarcoma 180 tumor-bearing mice

**DOI:** 10.1080/10717544.2019.1574938

**Published:** 2019-03-05

**Authors:** Li Gao, Shang Cai, Awei Cai, Yang Zhao, Tangbing Xu, Yan Ma, Yan Xu, Yuan Wang, Hao Wang, Yong Hu

**Affiliations:** a School of Food and Biological Engineering, Hefei University of Technology, Hefei, People’s Republic of China;; b Department of Bone Disease and Bone Tumors Surgery, First Affiliated Hospital of Anhui Medical University, Hefei, People’s Republic of China;; c Department of Pathology, The Second People’s Hospital of Hefei, Hefei, People’s Republic of China;; d Department of Orthopeadic Surgery, Fourth Affiliated Hospital of Anhui Medical University, Hefei, People’s Republic of China

**Keywords:** Cisplatin, implants, sustained release, PLGA, intratumoral chemotherapy

## Abstract

Cisplatin is the most commonly used antitumor drug in the chemotherapy of a variety of malignancies. However, the severe side effects and drug resistance limit its clinical application. The aim of this study was to develop PLGA-based cisplatin-loaded implants and evaluate the antitumor efficacy of continuous intratumoral chemotherapy with the implants. The cisplatin-loaded implants were prepared by the direct compression method and characterized regarding drug content, micromorphology, *in vitro* and *in vivo* drug release profiles. Furthermore, the antitumor activity of the implants was conducted in sarcoma 180 tumor-bearing mice. The SEM images showed smooth surface of the implants and the mean drug content of the tested implants was (37.7% ± 0.5%, w/w). Both *in vitro* and *in vivo* release profiles of the implants were characterized by initial burst release followed by the sustained-release of cisplatin. Intratumoral implantation of the cisplatin-loaded implants could effectively inhibit the tumor growth. Additionally, intratumoral chemotherapy with the implants significantly reduced the systemic toxicity compared with intravenous injection of cisplatin. It is worth noting that an increase in the dose of the implants led to a higher tumor suppression rate without additional systemic toxicity. These results demonstrated that cisplatin-loaded implants enhanced the antitumor efficacy and reduced the dose-related side effects in sarcoma 180 tumor-bearing mice.

## Introduction

Cancer is the leading cause of death worldwide in the 21st century. According to the GLOBOCAN 2018 estimates of cancer incidence and mortality produced by the International Agency for Research on Cancer (IARC), there are an estimated 18.1 million new cancer cases and 9.6 million cancer deaths in 2018 (Bray et al., 2018). Though progress in cancer therapy has significantly reduced cancer incidence and improved survival, cancer is still a major public health problem and the leading cause of death in China (Chen et al., 2016). The conventional systemic chemotherapy is the most commonly used methods of cancer therapy. However, intravenously administered anticancer drugs must overcome transport barriers before reaching the cancer site. As a result, only a small fraction of drugs could be transported into the tumor, higher systemic doses result in undesirable side effects to normal tissues (Weinberg et al., 2008).

Local chemotherapy with polymer-based drug delivery systems has been considered as an very promising method to improve treatment and minimize systemic side effects (Wolinsky et al., 2012). Moreover, local chemotherapy is highlighted as potential future solution for both prevention and treatment of locally recurrent cancers (Mahvi et al., 2018).

Cisplatin (CDDP) is widely used in the clinic as the standard treatment of various types of cancers. It triggers malignant cell death by interacting with nuclear DNA and inducing the apoptosis (Florea and Büsselberg, 2011; Arnesano et al., 2013; Dasari et al., 2014). Though cisplatin demonstrates positive effects in cancer therapy, the severe side effects limit the dose which can be administered, such as nausea and vomiting, myelosuppression, immunosuppression, nephrotoxicity, neurotoxicity, hearing loss, and gastrointestinal toxicity (Manohar and Leung, 2018; Shahid et al., 2018). In addition, the innate and acquired resistances to cisplatin strongly limit its clinical application (Amable, 2016). To maintain the efficiency of cisplatin and decrease its side effects, various cisplatin-loaded drug delivery systems have been widely investigated, including direct delivery of cisplatin, delivery of its pro-drugs and combination delivery (Yue and Cao, 2016).

In this study, we fabricated cisplatin-loaded implants using poly (lactic-co-glycolic acid) copolymer (PLGA) as the main polymer matrix by the direct compression method. The cisplatin-loaded implants were characterized in terms of micromorphology, drug content, *in vitro* and *in vivo* drug release profiles. Furthermore, the antitumor activity of the implants was conducted in sarcoma 180 tumor-bearing mice. The results showed that continuous intratumoral chemotherapy with the cisplatin-loaded implants inhibited tumor growth efficiently and reduced the dose-related systemic toxicity significantly in sarcoma 180 tumor-bearing mice.

## Materials and methods

### Reagents and animals

Cisplatin injection was purchased from Jiangsu Hengrui Medicine Co., Ltd. (Jiangsu, China). Cisplatin (purity 99.8%) was purchased from Kunming Guiyan Pharmaceutical Co., Ltd. PLGA (75:25 lactide/glycolide; inherent viscosity 0.21 dL/g) was generously provided by Hefei Zhongren Science and Technology Co., Ltd. (Anhui, China). Polyethylene glycol 4000 (PEG4000) was from Beijing Huiyou Chemical Co., Ltd (Beijing, China). Both RPMI-1640 medium and fetal calf serum were purchased from Hyclone (Logan, UT, USA). Ultra-pure water was obtained in a milli-Q system from EMD Millipore (Billerica, MA, USA). All other chemicals were of analytical grade.

The mouse sarcoma 180 cells were obtained from the Cell Bank of the Chinese Academy of Sciences (Shanghai, China). Healthy male Kunming mice (6 ∼ 8 weeks) were purchased from Experimental Animal Center of Anhui Medical University (Anhui, China). The mice were kept at constant temperature (23 °C ± 2 °C) and humidity (50 ± 5%) and had free access to clean food and water. All animal protocols were approved by the Ethics Committee in Animal Experimentation at Hefei University of Technology (Anhui, China) following the guidelines for Care and Use of Laboratory Animals.

### Preparation of cisplatin-loaded implants

The cisplatin-loaded implants were prepared by the direct compression method under sterile conditions. The dry powders containing 40% cisplatin, 50% PLGA and 10% PEG 4000 (w/w) were sieved through a 80-mesh screen and blended thoroughly. The mixture was further molded into cylindrical implants.

### Characterization of cisplatin-loaded implants

#### Determination of drug content of the cisplatin-loaded implants

Determination of drug content of the cisplatin-loaded implants was performed according to the methods described in the Pharmacopoeia of the People’s Republic of China (Chinese Pharmacopoeia Committee, 2015). Ten implants were selected and weighed individually. Each implant was grounded with a pestle and mortar and dissolved in 0.9% (w/v) sodium chloride. The mixture was transferred to a volumetric flask, and the residue was further dissolved in an ultrasonic water bath for 20 min. Then the suspension was filtered, and 20 μl of the filtrates was analyzed by high-performance liquid chromatography (HPLC). The actual drug content of each implant was calculated.

#### Scanning electron microscopy (SEM)

The implants were imaged using the Hitachi SU8020 scanning electron microscope to characterize the surface and cross-section morphology. The images were obtained at 5.0 kV accelerating voltage. Before imaging, the samples were placed on metal sample holders and coated with gold for 90 s at 20 mA using JEOL JFC-1600 auto fine coater. The surface and the cross-section morphologies of the implants were visualized at a magnification of 3000.

#### *In vitro* release assay

The *in vitro* release assay was performed using the rotating basket method on the dissolution apparatus. Twenty milligrams of cisplatin-loaded implants were placed in 300 ml 0.9% (w/v) sodium chloride. The rotating speed of the basket was set at 120 rpm, and the temperature of the release medium was maintained at 37 °C ± 0.5 °C. At the predetermined time points, 5 ml of the sample was withdrawn, filtered and analyzed by HPLC. Then 5 ml of fresh release medium was added back to the dissolution flask to maintain the constant sink condition. The measurement was performed in triplicate for each batch.

#### In vivo release assay

The *in vivo* release assay of the cisplatin-loaded implants was conducted by intratumorally implanting the implants into sarcoma 180 tumor-bearing mice. One implant was weighed and inserted into the center of the tumor. At 1, 5, 10, 15 and 20 days after implantation, the mice were euthanized by CO_2_ asphyxiation, and the cisplatin-loaded implant was retrieved, rinsed with deionized water, dried and stored at 4 °C until analysis. Three mice were used at each time point. The amount of drug in the residual implant was determined by HPLC. The *in vivo* cumulative release percentage of cisplatin was calculated as follows:

$$\text{Cisplatin}\;\text{release}\;\text{percentage}\left(\%\right)=\frac{\text{Initial}\;\text{cisplatin}\;\text{amount }-\text{ Residual}\;\text{cisplatin}\;\text{amount}}{\text{Initial}\;\text{cisplatin}\;\text{amount}\;}\times 100\%$$

### The HPLC method for determination of drug content in the cisplatin-loaded implants

The HPLC method was used to detect the content of cisplatin in the implants according to the Pharmacopoeia of the People’s Republic of China (Chinese Pharmacopoeia Committee, 2015). The HPLC system (Shimadzu, Japan) was equipped with two LC-15C pumps, an SPD-15C essential UV detector, and a CTO-15C essential column oven. The Waters Symmetry C18 column (4.6 × 250 mm, 5 µm particle size) was used as the analytical column and maintained at 25 °C in the column oven. The mixture of sodium 1-heptanesulfonate (0.003 mol/L) and sodium chloride solution (0.9%, w/v) was used as mobile phase and the flow rate was 1.5 ml/min. The injection volume was 20 µl and UV detection was performed at 220 nm.

### Antitumor efficacy of the cisplatin-loaded implants

#### Cell culture and sarcoma 180 mouse tumor model

The mouse sarcoma 180 cells were cultured in RPMI-1640 medium supplemented with 10% fetal calf serum. The cells were cultured at 37 °C in a humidified incubator in an atmosphere of 95% oxygen and 5% carbon dioxide. The cell suspension was adjusted to 1 × 10^7^ cells/ml and 100 µl of the suspension was injected subcutaneously into the armpit of a right anterior limb of each mouse (Gao et al., 2017). The *in vivo* studies were started when the tumor volume reached 100–200 mm^3^.

#### In vivo antitumor efficacy

Forty sarcoma 180 tumor-bearing mice weighing 33–35 g were randomly divided into four groups (*n* = 10 per group): (i) the negative control group without treatment (control group), (ii) tail vein injection of cisplatin solution at the dose of 25.75 mg/kg as positive control group (CDDP injection group), (iii) single intratumoral implantation of low-dose cisplatin-loaded implants at the dose of 25.75 mg/kg (CDDP implants-L group), (iv) single intratumoral implantation of high-dose cisplatin-loaded implants at the dose of 51.5 mg/kg (CDDP implants-H group). The dosage of cisplatin solution used in mice was calculated according to the clinical usage of cisplatin (100 mg/m^2^) for the chemotherapy of osteosarcoma (National Comprehensive Cancer Network, 2018). The hair near the solid tumor was shaved and the skin was disinfected with 70% ethanol. The implants were then inserted into the center of the tumor using the modified 17 gauge trochar provided by Hefei Zhongren Science and Technology Co., Ltd. The tumor volumes were measured every three days using a digital caliper and calculated by the formula: V (mm^3^) = (L × W^2^)/2, wherein the length (L) is the longest diameter and width (W) is the shortest diameter perpendicular to length (Dong et al., 2009). At the end point, mice were sacrificed and the tumors from each group were collected and weighed. Furthermore, the tumor suppression rate (TSR) was calculated using the formula TSR = (1 − Wt/Wc) × 100%, where Wt and Wc represent the mean final tumor weight of treated group and negative control group, respectively (Dong et al., 2013). When the tumor size reached 20 mm in any direction, it was considered as the humane endpoint (Mitchell et al., 2011).

To evaluate the toxicity after receiving cisplatin solution or cisplatin-loaded implants, body weight was measured throughout the whole therapy period. The daily general physical condition and mental active state of each mouse were observed. At the end of the experiment, blood samples were collected and examined hematologically using the automated cell counters. Moreover, serum biochemical analysis was carried out using the automatic chemistry analyzer (Pointcare 2Vi, Tianjin MNCHIP, China).

#### Histopathological studies

The histopathological analysis was carried out at the end of study (on day 22 after treatment). The mice were sacrificed and the organs (heart, liver, spleen, lung and kidney) and tumor tissues were isolated. Then, all the tissues were fixed in 10% neutral formalin solution and then dehydrated in a gradient ethanol series. The tissues were embedded in paraffin and sectioned at 4 μm thickness. Tissue sections were stained with hematoxylin and eosin for histopathological examination. The histological images were taken using an Olympus BX51 microscope system (Olympus Corporation, Tokyo, Japan).

### Statistical analysis

All data were expressed as mean ± standard deviation and analyzed using GraphPad Prism version 7.0 (GraphPad Software, Inc., La Jolla, CA, USA). One-way ANOVA of Tukey's multiple comparison tests was used to compare the mean of all experimental groups. The Kaplan–Meier log-rank test was used to compare survival between mice in different groups. *p* value less than .05 was considered as statistical significance.

## Results

### Preparation of cisplatin-loaded implants

The cisplatin-loaded implants were prepared by blending the mixture of cisplatin, PLGA and PEG4000 at a certain proportion. The mixture were further molded into solid cylinders with the average diameter of 0.9 mm and length of (1.97 ± 0.08) mm (Figure S1). In addition, the average weight of the tested implants was 3 mg and the mean actual drug content was (37.7% ± 0.5%,w/w) (*n* = 10).

### Micromorphology of cisplatin-loaded implants

**Figure 1.**
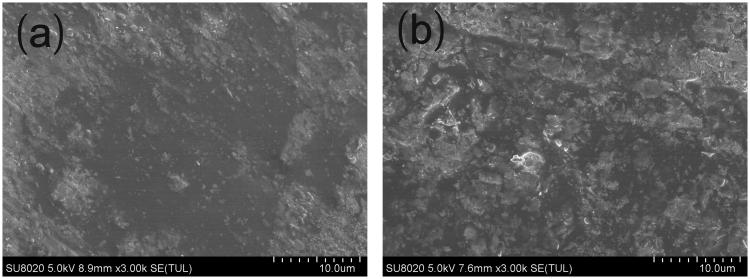
SEM picture of the cisplatin-loaded implants (magnification ×3000). (a) External surface of the implant, (b) Cross-section of the implant.

SEM was used to evaluate the microstructure of the cisplatin-loaded implants. The external surface of the implant was found to be smooth and homogenous (Figure 1(a)). Furthermore, the implant was cut with a scalpel to observe the internal morphology. The cross-section of the implant was a little rough but still homogenous in SEM (Figure 1(b)).

### In vitro and in vivo drug release from the implants

**Figure 2.**
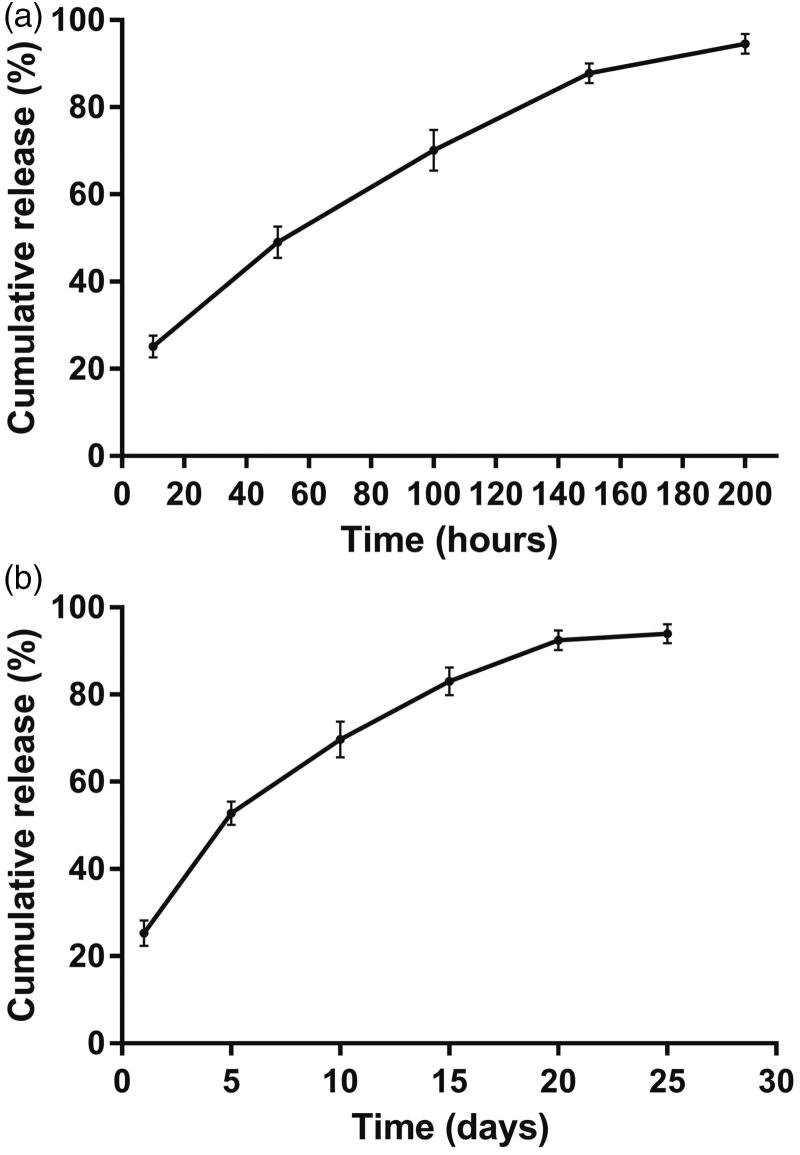
The release profiles of cisplatin-loaded implants. (a) The *in vitro* cumulative release profiles of cisplatin from the implants, (b) The *in vivo* cumulative release profiles of cisplatin from the implants. Data are shown as mean ± standard deviation (*n* = 6 for each time).

The *in vitro* cumulative release test was carried out in the release medium under suitable sink condition. The *in vitro* release profile was shown in Figure 2(a). Approximately 25% of the drug was released in the first 10 h. Subsequently, cisplatin was released from the implants almost at a constant rate. The mean cumulative release percentage reached 87.8% in a 150-hour period. As a whole, the final cumulative release reached an average of 95% within 200 h.

To gain the information of the *in vivo* release profile, the cisplatin-loaded implants were implanted intratumorally into the sarcoma 180 tumor-bearing mice and then the implants were collected on day 1, 5, 10, 15, 20, and 25, post implantation. The result was depicted in Figure 2(b). The cisplatin-loaded implants released 25.3% of the drug on day 1. Approximately 53% of the drug was released from the implants within 5 days. After that, cisplatin could be released steadily from the implants. The mean cumulative release percentage reached 94% on day 25.

### Antitumor efficacy of cisplatin-loaded implants

**Figure 3.**
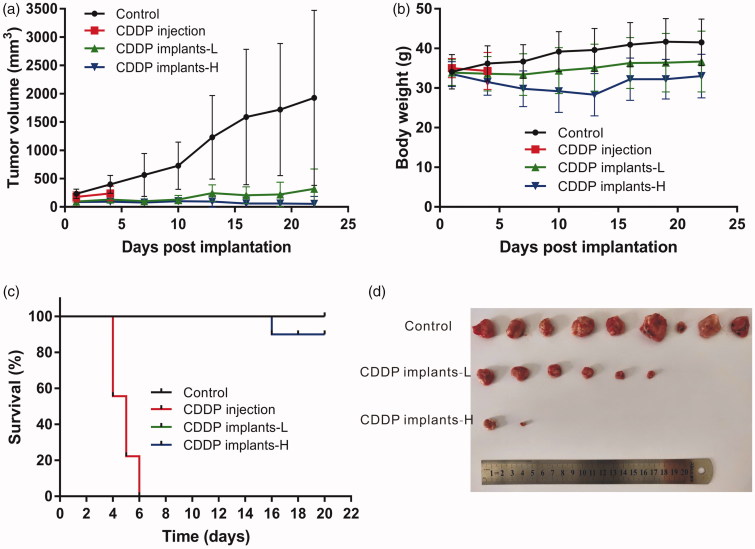
Antitumor efficacy of cisplatin-loaded implants on sarcoma 180 tumor-bearing mice. (a) Tumor growth curve of the sarcoma 180 tumor-bearing mice during the treatment period, (b) The average body weight of mice during the treatment period, (c) Kaplan–Meier curves for survival of sarcoma 180 tumor-bearing mice, (d) Picture of tumors dissected from the mice at the end of experiment.

The evaluation of antitumor activity was conducted in sarcoma 180 tumor-bearing Kunming mice. The tumor growth curve was shown in Figure 3(a), the tumor had grown rapidly in control group. However, tumor shrinkage was observed in one mouse of control group on day 10 after treatment. Intratumoral implantation of the cisplatin-loaded implants inhibited tumor growth significantly. Moreover, high-dose cisplatin-loaded implants resulted in more significant tumor inhibition compared with other groups. It is worth noting that 40% of mice in CDDP implants-L group and 70% of mice in CDDP implants-H group exhibited complete tumor regression at the end of the experiment.

During the experiment period, the body weights of mice in control group and CDDP implants-L group increased slowly. However, the mean body weight in CDDP implants-H group decreased slowly and reached its lowest point on day 13 post implantation. And then the mice in CDDP implants-H group showed increased body weights during the observation period (Figure 3(b)).

At the end of the experiment, mice were sacrificed and tumors dissected from the mice were weighed to calculate the TSR (Table S1). The mean final tumor weight of control group was significantly higher than CDDP implants-L and CDDP implants-H groups. Furthermore, the mean tumor weight of CDDP implants-H group was significantly lower than CDDP implants-L group. The value of TSR of CDDP implants-H group (84%) was greater than that in CDDP implants-L group (58%).

**Table 1.** The hematological parameters of sarcoma 180 tumor-bearing mice (*n* = 10).

	Control	CDDP implants-L	CDDP implants-H
Erythrocyte (×10^12^/L)	4.14 ± 0.83	5.7 ± 1.0	4.2 ± 1.1
Hemoglobin (g/L)	63.6 ± 12.0	92.9 ± 15.8	66.4 ± 15.6
Hematocrit (%)	16.5 ± 2.6	23.6 ± 4.2	16.5 ± 4.5
MCV (fL)	40.3 ± 2.1	41.4 ± 2.5	39.9 ± 1.4
MCHC (g/L)	383 ± 14.2	394.2 ± 14.7	405.2 ± 13.3
Leukocyte (×10^9^/L)	3.48 ± 1.13	4 ± 0.9	2.9 ± 1.5
Granulocyte (×10^9^/L)	1.2 ± 0.5	1.3 ± 0.6	0.8 ± 0.7
Lymphocyte (×10^9^/L)	1.3 ± 0.2	1.9 ± 0.5	1.3 ± 1.0
Monocyte (×10^9^/L)	1.0 ± 0.6	0.8 ± 0.2	0.7 ± 0.4
Platelet (×10^9^/L)	487.6 ± 190.2	457.6 ± 131.8	445.4 ± 65.6

CDDP implants-L is cisplatin-loaded implants at the dose of 25.75 mg/kg; CDDP implants-H is cisplatin-loaded implants at the dose of 51.5 mg/kg.MCV: mean corpuscular volume; MCHC: mean corpuscular hemoglobin concentration.

The survival curve was shown in Figure 3(c), the mice in control group and CDDP implants-L group (25.75 mg/kg) survived until the end of the experiment. In CDDP implants-H group (51.5 mg/kg), one death was observed on day 16 after treatment. All mice died within 6 days after receiving intravenous administration of cisplatin.

The parameters of hematological examination were summarized in Table 1. We did not observe the difference of blood cell counts in cisplatin-loaded implants treated groups compared with the control group. Furthermore, the serum biochemical examination showed that there was no statistically significant difference between the cisplatin-loaded implants treated groups and control group (Table 2).

**Table 2.** The biochemical analyses of serum from sarcoma 180 tumor-bearing mice (*n* = 10).

	Control	CDDP implants-L	CDDP implants-H
STP (g/L)	67.73 ± 6.6	64.93 ± 1.81	58.63 ± 2.57
Albumin (g/L)	32.13 ± 2.39	32.10 ± 1.04	29.57 ± 1.40
Globulin (g/L)	35.60 ± 6.32	32.80 ± 0.80	29.07 ± 1.65
TB (umol/L)	4.85 ± 0.96	5.81 ± 1.55	3.78 ± 1.05
ALT (U/L)	50.00 ± 31.60	30.33 ± 9.29	46.00 ± 4.36
AST (U/L)	323.00 ± 199.73	197.00 ± 144.11	124.00 ± 20.95
BUN (mmol/L)	5.65 ± 2.20	4.09 ± 0.35	5.22 ± 0.87
Glucose (mmol/L)	6.12 ± 0.92	5.94 ± 0.40	6.07 ± 0.91

STP: serum total protein; TB: total bilirubin; ALT: alanine aminotransferase; AST: Aspartate aminotransferase; BUN: blood urea nitrogen.

**Figure 4.**
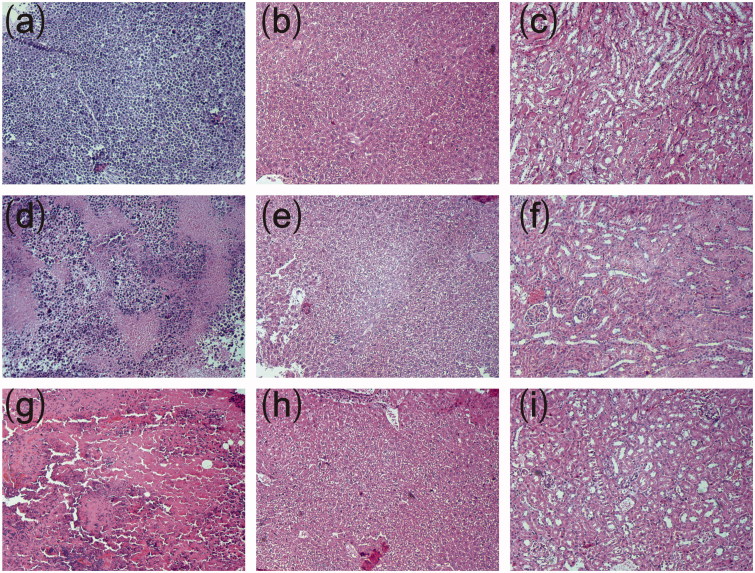
Typical histopathological images of tumors and major organs of sarcoma 180 tumor-bearing mice (magnification ×100). (a) Histopathological image of tumor without treatment, (b) Histopathological image of liver without treatment, (c) Histopathological image of kidney without treatment, (d) Histopathological image of tumor treated with low-dose cisplatin-loaded implants, (e) Histopathological image of liver treated with low-dose cisplatin-loaded implants, (f) Histopathological image of kidney treated with low-dose cisplatin-loaded implants, (g) Histopathological image of tumor treated with high-dose cisplatin-loaded implants, (h) Histopathological image of liver treated with high-dose cisplatin-loaded implants, (i) Histopathological image of kidney treated with high-dose cisplatin-loaded implants.

Representative histopathological photographs of tumor tissues and major organs were presented in Figure 4. The tumor from control group was filled with viable tumor cells while those from cisplatin-loaded implants treated groups exhibited evident necrotic areas mixed with cellular debris. Larger areas of necrosis were observed in tumor tissues treated with high-dose cisplatin-loaded implants. Histopathological analyses of heart, liver, spleen, lung and kidney tissues were carried out to evaluate the systemic toxicity of cisplatin-loaded implants. We did not find obvious necrosis in the tissues from both CDDP implants-L group and CDDP implants-H group.

## Discussion

Cisplatin is a platinum-containing drug. It is currently used in the treatment of a variety of cancers including testicular, ovarian, bladder, head and neck, esophageal, small and non-small cell lung cancer, breast, cervical, stomach, prostate cancers, Hodgkin’s and non-Hodgkin’s lymphomas, neuroblastoma, sarcoma, multiple myeloma, melanoma, and mesothelioma (Dasari et al., 2014; Manohar and Leung, 2018). Nowadays there are two problems associated with cisplatin usage in the clinic: resistance and toxicity. The molecular mechanisms of resistance to cisplatin involves the alteration of DNA repair, reduced cellular accumulation of drug and cytosolic inactivation of drug (Galluzzi et al., 2014; Amable, 2016). Additionally, patients receiving cisplatin treatment have experienced severe and diverse side effects that limited the clinic dose can be administered (Florea and Büsselberg, 2011). In this study, we developed cisplatin-loaded implants directly targeting at tumor site and aimed to maximize the antitumor efficacy of cisplatin while reducing the treatment-related side effects.

The cisplatin-loaded implants were prepared by the direct compression method and PLGA was used as the main excipient of the implants. PLGA is a copolymer of poly(lactic acid) (PLA) and poly(glycolic acid) (PGA) that have been widely used in the drug delivery systems because of its good biocompatibility, degradability, and minimal toxicity in physiological environments (Xu et al., 2017; Chereddy et al., 2018). PEG 4000 was the other excipient of the cisplatin-loaded implants. PEG polymer was characterized by low melting point, low toxicity, compatibility and hydrophilicity. The addition of PEG can promote dissolution and increase the release rate of the drug from the implant by promoting water diffusion (El-Badry et al., 2009; Cheng et al., 2010; Wang et al., 2015).

Ten cisplatin-loaded implants were selected and tested the drug content by HPLC complied with the method described in the Pharmacopoeia of the People’s Republic of China. The mean value of actual drug content of the tested implants was (37.7 ± 0.5) % which was close to the label claim of the implants (40%, w/w). The low standard deviation (0.5%) of the drug content revealed the good content uniformity of the implants. Furthermore, the SEM image of the implants demonstrated that the drug distributed homogenously in the formulation, indicating that cisplatin and the excipients were mixed sufficiently in the fabrication process.

The results of *in vitro* and *in vivo* drug release indicated that the cisplatin-loaded implants exhibited initial burst effect followed by sustained-release of cisplatin. More than 20% of drug released from the implants within one day both *in vitro* and *in vivo*. The initial burst release may be due to the fast dissolution and diffusion of cisplatin from the surface of the implants. The implants released a large amount of drug early to rapidly reach the therapeutic concentration at the tumor target. Then the sustained-release of drug provided a maintenance dose to remain the effective drug level (Weinberg et al., 2008). The drug release of the implants depends on the environmental conditions and the physicochemical properties of the drug and polymer. Additionally, the shape, size and drug content of the implants also influence the drug release profiles from the implants (Li et al., 2010; Solano et al., 2013). The optimal drug delivery profiles will maximize the treatment success of the implants.

The antitumor efficacy of the cisplatin-loaded implants was investigated using a sarcoma 180 murine tumor model. We found that intratumoral chemotherapy with the implants inhibited tumor growth efficiently. The TSR value increased when higher doses of cisplatin-loaded implants were given because a large amount of cisplatin released from the implants and accumulated in the tumor site, thus resulting in strong suppression of tumor growth. It is well known that reduced accumulation of cisplatin is one of the molecular mechanisms of cisplatin resistance. Tissue platinum concentration are correlated with the reduction of the tumor (Amable, 2016). Intratumoral implantation of cisplatin-loaded implants may be an alternative method to overcome resistance to cisplatin.

The fast development of cell resistance and the presence of acute side effects are the main drawbacks of platinum drugs (Arnesano et al., 2013). In this study, intravenous injection of cisplatin caused 100% mortality in mice. However, the mice receiving cisplatin-loaded implants survived till the end of the experiment except one death was observed in high-dose implants (double dose of cisplatin injection) treated mice. The histopathological evaluation of tumor tissues confirmed the antitumor activity of the implants. Intratumoral implantation of cisplatin-loaded implants induced tumor cell death directly and higher-dose implants resulted in more severe tumor cell destruction. The results of hematological and blood biochemical examination revealed that the cisplatin-loaded implants did not cause systemic toxicity compared with control group. In addition, the histopathological analyses of tissues of heart, liver, spleen, lung, and kidney revealed no significant damage to the major organs of mice treated with the implants.

Several studies have reported that intratumoral injection of cisplatin-loaded drug delivery systems effectively enhanced antitumor efficiency (Shikanov et al., 2011; Zong et al., 2015; Yang et al., 2016; Chang et al., 2018). In this paper, intratumoral chemotherapy with the cisplatin-loaded implants delivered cisplatin directly to the tumor site and released the drug locally for a long time. The prolonged exposure of cisplatin to cancer cells produced a long period of tumor growth inhibition and minimized the systemic toxicities.

## Conclusion

In this study, we prepared PLGA-based cisplatin-loaded implants by direct compression method. The results of drug content and SEM indicated that cisplatin was homogeneously dispersed in the polymeric matrix. Both *in vitro* and *in vivo* drug release profiles of the implants were characterized by high initial burst release followed by sustained release of cisplatin. Intratumoral delivery of cisplatin-loaded implants demonstrated significant antitumor efficacy on sarcoma 180 tumor-bearing mice with minimized systemic toxicity. We conclude that continuous intratumoral chemotherapy with cisplatin-loaded implants have the potential role to be used as a new method to treat cancer.

## Supplementary Material

Table_S1.The_TSR_of_control_group_and_cisplatin-loaded_implants_treated_groups.doc

Figure_S1._Macroscopic_picture_of_the_entire_cisplatin-loaded_implants.jpg
