# Supplementary material for: The improved antitumor efficacy of continuous intratumoral chemotherapy with cisplatin-loaded implants for the treatment of sarcoma 180 tumor-bearing mice
Source: Drug Deliv. 2019 Mar 5;26(1):208–15. doi: 10.1080/10717544.2019.1574938 (PMC6407574; doi:10.1080/10717544.2019.1574938)
Supplement: Table_S1.The_TSR_of_control_group_and_cisplatin-loaded_implants_treated_groups.doc [file IDRD_A_1574938_SM3855.doc]

**Table S1.** The TSR of control group and cisplatin-loaded implants treated groups

| **Groups** | **Mean Tumor Weight (g)** | **TSR (%)** |
| --- | --- | --- |
| Control | 1.9 ± 1.2 |  |
| CDDP implants-L | 0.8 ± 0.4 | 58 |
| CDDP implants-H | 0.3 ± 0.2 | 84 |

**Notes**: CDDP implants-L is cisplatin-loaded implants at the dose of 25.75 mg/kg;

CDDP implants-H is cisplatin-loaded implants at the dose of 51.5 mg/kg.

**Abbreviations:** TSR, tumor suppression rate; CDDP, cisplatin
