# Supplementary figures and images for: The improved antitumor efficacy of continuous intratumoral chemotherapy with cisplatin-loaded implants for the treatment of sarcoma 180 tumor-bearing mice
Source: Drug Deliv. 2019 Mar 5;26(1):208–15. doi: 10.1080/10717544.2019.1574938 (PMC6407574; doi:10.1080/10717544.2019.1574938)

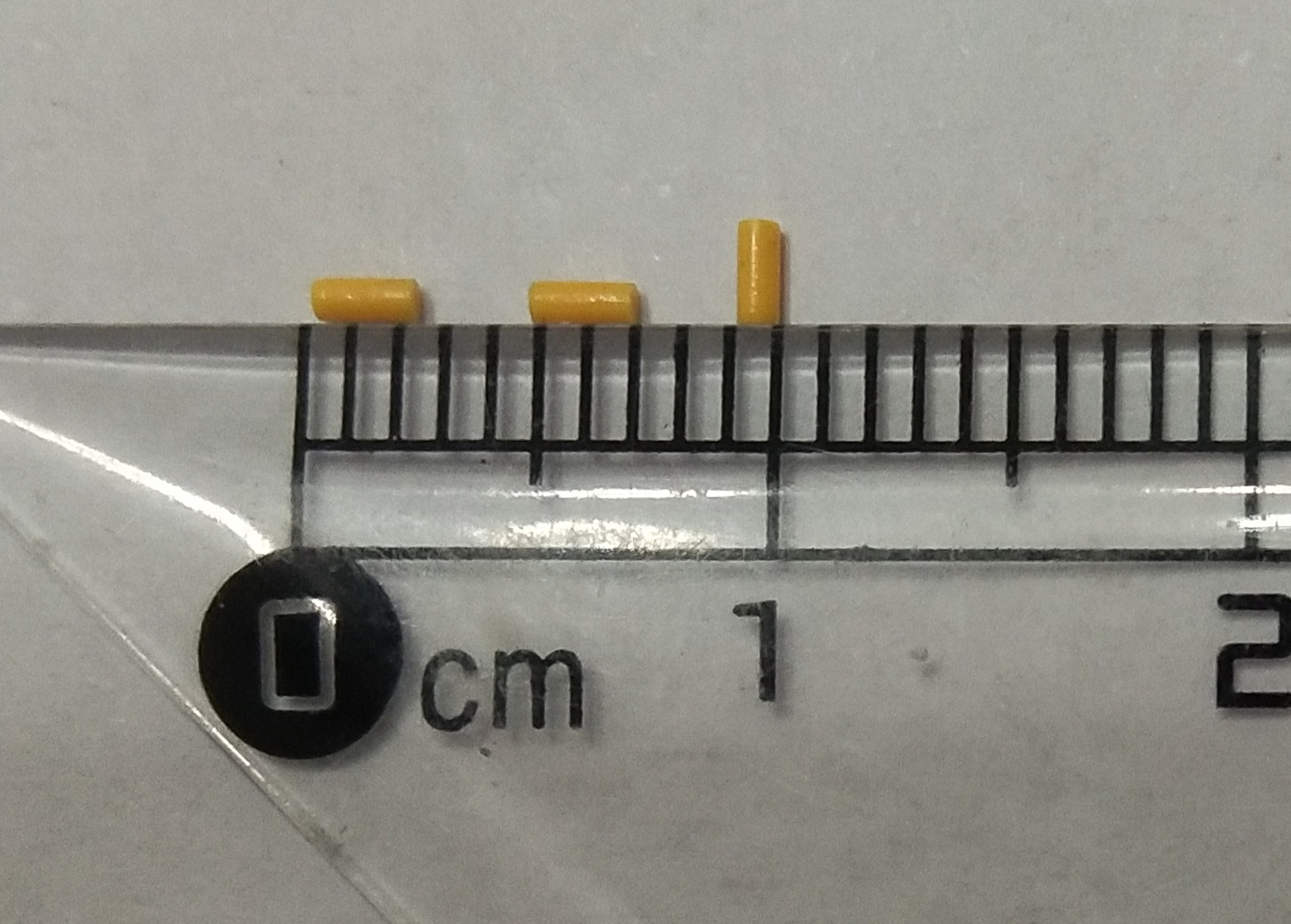

Supplement: Figure_S1._Macroscopic_picture_of_the_entire_cisplatin-loaded_implants.jpg [file IDRD_A_1574938_SM3854.jpg]
